# Petrobactin Protects against Oxidative Stress and Enhances Sporulation Efficiency in Bacillus anthracis Sterne

**DOI:** 10.1128/mBio.02079-18

**Published:** 2018-11-06

**Authors:** Ada K. Hagan, Yael M. Plotnick, Ryan E. Dingle, Zachary I. Mendel, Stephen R. Cendrowski, David H. Sherman, Ashootosh Tripathi, Philip C. Hanna

**Affiliations:** a Department of Microbiology and Immunology, University of Michigan, Ann Arbor, Michigan, USA; b Life Sciences Institute, Department of Medicinal Chemistry, University of Michigan, Ann Arbor, Michigan, USA; c Life Sciences Institute, Department of Medicinal Chemistry, Department of Chemistry, Department of Microbiology and Immunology, University of Michigan, Ann Arbor, Michigan, USA; University of Chicago

**Keywords:** *Bacillus anthracis*, LAESI-MS, oxidative stress, petrobactin, siderophore, sporulation

## Abstract

Bacillus anthracis is a Gram-positive bacillus that under conditions of environmental stress, such as low nutrients, can convert from a vegetative bacillus to a highly durable spore that enables long-term survival. The sporulation process is regulated by a sequential cascade of dedicated transcription factors but requires key nutrients to complete, one of which is iron. Iron acquisition by the iron-scavenging siderophore petrobactin is required for vegetative growth of B. anthracis under iron-depleted conditions and in the host. However, the extent to which petrobactin is involved in spore formation is unknown. This work shows that efficient *in vitro* sporulation of B. anthracis requires petrobactin, that the petrobactin biosynthesis operon (*asbA* to *-F*) is induced prior to sporulation, and that the siderophore itself associates with spores. Petrobactin is also required for oxidative stress protection during late-stage growth and for wild-type levels of sporulation in sporulation medium. Sporulation in bovine blood was found to be petrobactin dependent. Collectively, the *in vitro* contributions of petrobactin to sporulation as well as growth imply that petrobactin may be required for B. anthracis transmission via the spore during natural infections, in addition to its key known functions during active anthrax infections.

## Abstract (precis)

Bacillus anthracis causes the disease anthrax, which is transmitted via its dormant, spore phase. However, conversion from bacillus to spore is a complex, energetically costly process that requires many nutrients, including iron. B. anthracis requires the siderophore petrobactin to scavenge iron from host environments. We show that, in the Sterne strain, petrobactin is required for efficient sporulation, even when ample iron is available. The petrobactin biosynthesis operon is expressed during sporulation, and petrobactin is biosynthesized during growth in high-iron sporulation medium, but instead of being exported, the petrobactin remains intracellular to protect against oxidative stress and improve sporulation. It is also required for full growth and sporulation in blood (bovine), an essential step for anthrax transmission between mammalian hosts.

## INTRODUCTION

Bacillus anthracis is a Gram-positive, spore-forming bacillus that causes the disease anthrax. In humans, anthrax can manifest in four ways, depending on the route of exposure to B. anthracis spores: cutaneous, inhalational, gastrointestinal, or injectional (1, 2). Following aerosol exposure, the spores, a metabolically dormant form of B. anthracis, are taken up by antigen-presenting cells (APCs) such as macrophages and dendritic cells (3, 4). While associated with APCs, a set of small molecules from the host initiate germination of spores into vegetative bacilli (3). The bacilli rapidly initiate cellular functions and within 30 min begin transcription and translation of required proteins, including the toxins that both enable escape from the APC and cause anthrax pathologies (5). If the APC is in transit to proximal lymph nodes when escape occurs, the bacilli are released directly into the blood or lymph to replicate, quickly reaching titers greater than 10^8^ CFU/ml (6, 7).

The B. anthracis spore is the infectious particle in anthrax transmission. Bacterial spores are dormant structures that enable the bacterium to survive harsh conditions, including nutrient deprivation, extreme temperatures, radiation, and desiccation (6, 8–11). As nutrients diminish and cell density increases, environmental sensors initiate a cascade of transcriptional regulators to activate genes whose products construct a spore from both the inside out and the outside in (12–14). Most of the research describing sporulation has been conducted in Bacillus subtilis, with B. anthracis containing close homologues to most B. subtilis sporulation system genes. These are described in brief here (see reference 12 for a recent review).

The first morphological change observed during sporulation is asymmetric division of a bacillus into the mother cell and prespore compartments, which is initiated by phosphorylation of the transcriptional regulator Spo0A and activation of the sporulation-specific sigma factor σ^H^ (12, 16, 17). The next step in transcriptional regulation is the compartmentalized activation of two early sporulation sigma factors, σ^F^ and σ^E^, in the prespore and mother cell, respectively (12). A suite of σ^F^- and σ^E^-dependent proteins enable engulfment of the prespore by the mother cell in the second major morphological change (12, 18). Final maturation of the spore is regulated by the prespore-specific σ^G^ and the mother-cell-specific σ^K^ (12, 19, 20). When completed, the spore structure is composed of a dehydrated core, containing the genome and silent transcriptional and translational machinery, surrounded by an inner membrane, a layer of modified peptidoglycan known as the cortex, an outer membrane, a proteinaceous spore coat, and, for B. anthracis, the exosporium (9, 12, 14).

Sporulation is an energetically costly process. While sporulation is initiated by nutrient depletion, efficient sporulation still requires access to many nutrients, including large amounts of iron (1.5 to 2 mM) (21, 22). Iron is required as a cofactor for enzymes requiring electron transfer, such as those involved in environmental sensing, ATP synthesis, and the tricarboxylic acid cycle (23). To scavenge iron from the environment during low iron availability, many bacteria can synthesize small molecules called siderophores. Under iron-replete conditions, however, siderophores and other iron acquisition systems are repressed by the ferric uptake repressor Fur or a similar system. Fur is a dual iron- and DNA-binding protein. In the iron-bound form, Fur tightly binds sequences known as Fur boxes, thus repressing transcription of any downstream genes. Low-iron stress causes the iron to be shunted from Fur to essential cellular processes, which derepresses Fur-regulated genes, allowing for expression of iron acquisition systems (24, 25).

While iron is essential for many bacterial processes, excess free iron is toxic to the cell. Countering potential iron toxicities requires dedicated proteins to prevent the formation of superoxide radicals via participation of iron in the Fenton reaction. Iron in B. anthracis is sequestered by ferritins, the mini-ferritin DPS, and superoxide dismutases (26, 27). These proteins contribute to iron storage in B. anthracis spores (∼10 µM), which is presumed to be required for outgrowth from the spore under iron-limiting conditions (e.g., within an APC endosome), until active iron acquisition systems can be expressed 1 to 2 h following germination (28, 29). One such system is the siderophore petrobactin, whose biosynthetic machinery is encoded by the *asb* operon and is induced within 2 h of germination (28, 30).

B. anthracis has three known active iron acquisition systems: the two siderophores petrobactin and bacillibactin and a heme acquisition system. Petrobactin is required for growth in macrophages and virulence in a murine inhalational anthrax model (30, 31). Previous studies have elucidated much about petrobactin use in B. anthracis, including defining the biosynthetic pathway for petrobactin (the *asb* operon), the petrobactin-iron complex receptor (FhuA), import permeases (FpuB/FatC/FatD), ATPases (FpuC/FatE), and the petrobactin exporter (ApeX) (15, 30, 32–34). However, previous studies have also suggested that the *asb* operon may be regulated by environmental conditions other than iron (35, 36). In the present work, we investigated whether petrobactin-dependent iron acquisition plays a role in aspects of B. anthracis Sterne spore biology and the associated regulation of *asb*.

## RESULTS

### Petrobactin is required for sporulation but not germination.

**FIG 1:**
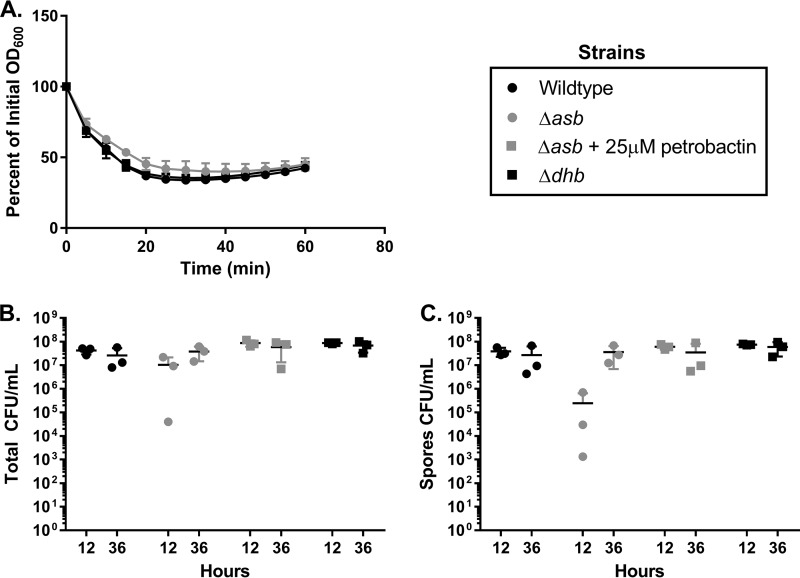
*asb* mutant spores germinate but fail to sporulate efficiently. (A) Wild-type, *dhb* mutant, and *asb* mutant spores were inoculated in IDM plus 1 mM inosine at a starting OD_600_ of between 0.25 and 0.5. The OD_600_ was measured every 5 min for 1 h. Data are presented as a percentage of the initial OD_600_ and are representative of *n* = 3 experiments. (B and C) Overnight cultures of bacilli of the wild type, the *asb* mutant ± 25 µM petrobactin, and the *dhb* mutant were inoculated in 3 ml of ModG medium and incubated at 37°C with shaking. At 12 and 36 h postinoculation, the total (B) and spore (C) CFU/ml were determined by serial dilution and plating. Data were compiled from three independent experiments.

Spores cannot be infectious particles without first germinating to the vegetative state, so to begin evaluating the role of petrobactin in spore biology, initial experiments investigated the effect of petrobactin on germination under low-iron conditions. To observe germination kinetics, spores of wild-type B. anthracis Sterne, an *asb* (petrobactin-null) mutant strain, and a *dhb* (bacillibactin-null) mutant strain were incubated in iron-depleted medium supplemented with 1 mM inosine (IDM+I) for 1 h. The *asb* mutant did not display any defect in germination relative to either wild-type or *dhb* mutant spores (Fig. 1A).

To further explore our hypothesis that petrobactin plays a role in spore biology, we tested the ability of an *asb* mutant strain and a *dhb* mutant strain to sporulate relative to the wild type. The timing of B. anthracis sporulation ranges from 5 h (28) to 2 weeks (37), depending on growth conditions. Based on preliminary experiments (data not shown), CFU/ml were enumerated at 12 and 36 h of growth in sporulation medium to determine total and sporulated counts. Despite an abundance of ferrous iron (1.7 mM) in the medium and growth to 10^7^ CFU/ml (Fig. 1B), less than 10^6^ CFU/ml (∼10%) of the *asb* mutant strain population had sporulated at 12 h postinoculation (Fig. 1C). That was nearly 2 log fewer spores than the wild-type and *dhb* mutant strains, whose spore populations exceeded 10^7^ CFU/ml at 12 h postinoculation (Fig. 1C). This defect in sporulation by the *asb* mutant strain was not observed at 36 h postinoculation, suggesting a delayed compensation mechanism. As a control, the defect was rescued at 12 h by supplementing the *asb* mutant strain with 25 µM purified petrobactin at inoculation (Fig. 1B and C). Since sporulation of the *asb* mutant strain can be complemented in *trans* with purified petrobactin, these data suggest that petrobactin is biosynthesized and that the *asb* operon is expressed prior to spore formation under this growth condition, despite the presence of high iron levels.

### The *asb* operon is transcribed and translated during late-stage growth and early sporulation.

**FIG 2:**
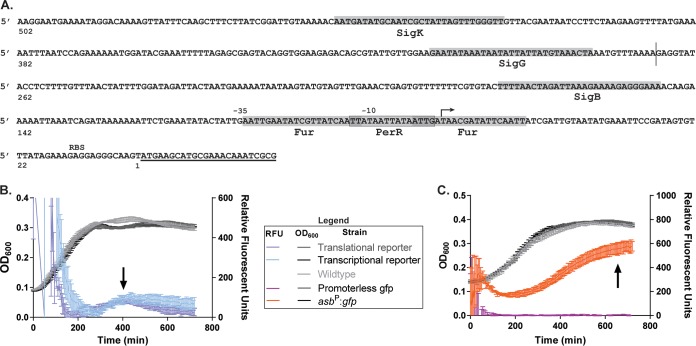
The *asb* transcriptional and translational fluorescent reporters illuminate expression during late-stage growth. (A) Schematic of putative transcriptional regulator binding sites (shaded regions) upstream of *asbA* (underlined). The bent arrow denotes the transcriptional start site in IDM (unpublished data [61]), and the vertical line indicates the start of the primer for the plasmid-based reporter. (B and C) Wild-type and fluorescent reporters were inoculated into ModG (plus 10 μg/ml chloramphenicol as needed) at a starting OD_600_ of 0.05. Growth (left axis [OD_600_]) and relative fluorescence units (right axis) were measured every 5 min for 12 h. Data are representative of three independent experiments. Black arrows indicate late-stage expression of the *asb* operon (B) genomic-based *asb* transcription and translation *gfpmut3*α fusion reporters and (C) plasmid-based promoterless *gfpmut3*α and *asb*::*gfpmut3*α transcriptional reporters.

To understand how *asb* might be expressed despite iron levels capable of suppressing expression during vegetative growth, we used the Database of Transcriptional Regulation in Bacillus subtilis (DBTBS) prediction tool to search the 500 bp upstream of *asbA* for putative sigma factor binding sites (38). There were potential consensus binding sites for two sporulation-specific sigma factors (σ^G^ and σ^K^), the general stress transcription factor σ^B^, and the oxidative stress response regulator PerR encoded in this region, all identified with at least 95% confidence (Fig. 2A). This suggests that an alternative regulation system or systems may be active during sporulation and could be responsible for the petrobactin-dependent sporulation phenotype. To characterize expression of the operon, fluorescent reporters were generated. Two reporter constructs for the *asb* operon, one transcriptional and one translational, were generated by fusing the 500 bp upstream of *asb* and the first eight codons of *asbA* (Fig. 2A, underlined)—either separated by a ribosomal binding site (transcriptional) or directly (translational)—to the green fluorescent protein (GFP) allele *gfpmut3*α (39) To facilitate wild-type-like expression of the reporters, each was inserted on the B. anthracis genome immediately downstream of the *asbF* transcriptional terminator by allelic exchange.

To measure reporter expression, strains of the transcriptional and translational reporters, along with the wild-type strain, were grown in sporulation medium with shaking for 12 h, and the optical density at 600 nm (OD_600_) and GFP fluorescence were measured every 5 min. The transcriptional and translational reporter strains both grew identically to the wild-type strain (Fig. 2B), and the calculated relative fluorescence units (RFU) indicate that *asb* is both transcribed and translated during stationary-phase growth in sporulation medium (Fig. 2B, black arrow). Despite correcting for the fluorescence of both medium alone and nonfluorescent cells, early time points in our experimental setup always displayed initial high fluorescence. We believe this could be due to either upregulation of the *asb* operon following transition to a new medium or refraction of fluorescence against the wells due to low cell density. Because our interests lie in the late stages of growth, the *y* axis (showing fluorescence values) is truncated at 600 RFU to better show the peak in stationary-phase fluorescence.

After observing that the *asb* operon is expressed during late-stage growth in sporulation medium, we next sought to determine if any of the predicted sporulation sigma factors were required for expression of expression of *asbA* to -*F* (*asbA–F*). Here, we used plasmid-based transcriptional reporter constructs where the 260 bp upstream of *asbA* (Fig. 2A, vertical line) were fused to *gfpmut3*α, cloned into the pAD123 expression vector, and expressed in a wild-type B. anthracis Sterne background. This construct lacks the predicted binding sites for sporulation-specific sigma factors σ^G^ and σ^K^ but retains predicted binding sites for Fur, σ^B^, and PerR (Fig. 2A).

To measure expression of *asbA–F* by this construct, the wild-type strain, the transcriptional reporter strain, and a promoterless *gfpmut3*α strain were grown in sporulation medium as described, and the RFU were similarly calculated. Overall growth kinetics were similar, and the 260-bp *asb* promoter was sufficient for *a*s*b* transcription during late-stage growth (Fig. 2C, black arrow). The observed increase in RFU for Fig. 2B versus Fig. 2C is likely an artifact from increased copy numbers of plasmid-based reporters. Together these data suggest that the high iron levels in the sporulation medium do not fully repress the *asb* operon by Fur and that sporulation-specific sigma factors are not required for expression of *asbA–F* during these conditions.

**FIG 3:**
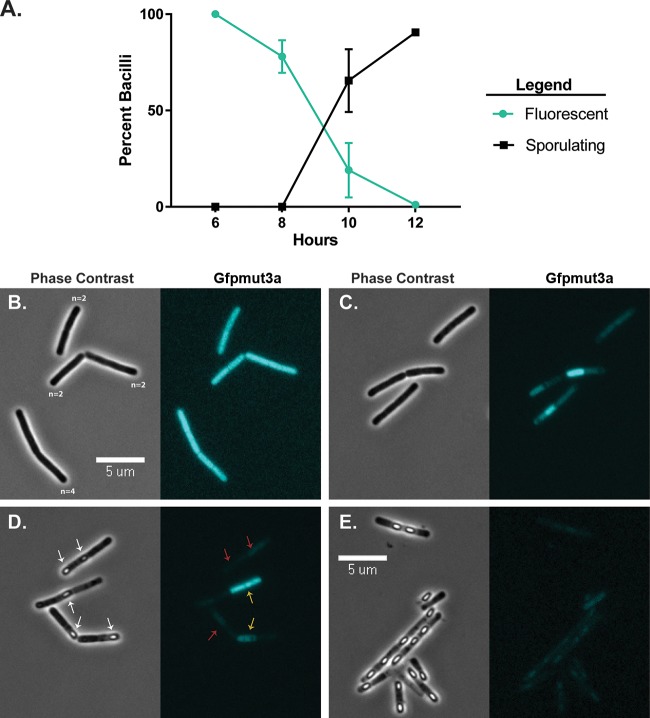
Translation of *asb* shuts down during late-stage growth while sporulation occurs. The *asb* translational reporter strain was grown in ModG sporulation medium with both phase-contrast and Gfpmut3α fluorescent micrographs taken at 6, 8, 10, and 12 h of growth, and the bacteria were scored for fluorescence and sporulation. (A) Pooled data from two replicates of the percentage of bacilli fluorescent and/or sporulating over time. (B to E) Representative phase-contrast and fluorescence images from each time point. (B) Six hours. The values 2 and 4 for *n* indicate the number of bacilli counted per chain. (C) Eight hours. (D) Ten hours. Shown are examples of scoring for phase-bright spores (white arrows), fluorescent (yellow arrows) and nonfluorescent (red arrows). (E) Twelve hours.

Given these conclusions, we next wanted to better understand the population dynamics and kinetics for *asb* expression relative to sporulation. The chromosome-based translational reporter and wild-type strains grown in sporulation medium were imaged with phase-contrast and fluorescence microscopy at 6, 8, 10, and 12 h postinoculation. Individual bacilli were scored for Gfpmut3α expression (positive is at least 1.4× above background fluorescence) and sporulation (if they contained phase-bright spores). (Representative images are shown in Fig. 3B to E; for the wild type, see Fig. S1.) At 6 h of growth, 100% of the translational reporter cells were fluorescent, thus expressing the *asb* operon (Fig. 3A and B). The number of fluorescent bacilli decreased over time, with 80% of the population expressing *asb* at 8 h of growth and only 20% at 10 h of growth (Fig. 3A, C, and D). No bacteria were scored as fluorescent at the 12-h time point (Fig. 3A and E). Phase-bright spores were not observed until 10 h postinoculation, at which point spores were present in 65% of bacilli (Fig. 3A and D). At 12 h postinoculation, 90% of the population were either sporulating or mature, free spores (Fig. 3A and E). Together with the data from Fig. 2B and C, these data indicate that *asb* expression peaks and terminates before maturation to phase-bright spores and likely before the onset of sporulation (especially given the long half-life of Gfpumut3α).

These observations confirm that *asb* expression does not require sporulation-specific sigma factors during sporulation (Fig. 2C), particularly since both σ^G^ and σ^K^ are active during the later stages of sporulation, by which point fluorescence has markedly declined—probably due to either degradation or protein dilution due to cell division. So, while petrobactin does not appear to be required for the process of sporulation vis a vis sporulation-specific regulation of *asb*, it is required for efficient sporulation and since cell stress precedes sporulation, *asb* expression may be induced by a stress response regulator such as σ^B^ or PerR.

### During sporulation, petrobactin is not exported, remains associated with the spore, and is protective against oxidative stress.

**FIG 4:**
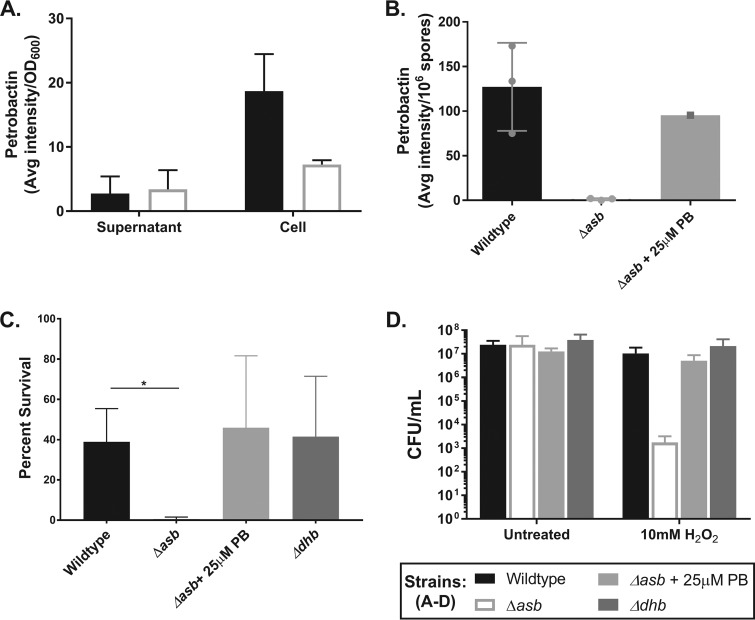
Petrobactin has an intracellular role to protect against oxidative stress and associates with the B. anthracis spore. (A) LAESI-MS analysis of petrobactin in the supernatants and cell pellets of wild-type and *asb* mutant strains grown in sporulation medium for 12 h. Data are presented as counts per OD_600_ unit and were pooled from three independent experiments. (B) LAESI-MS analysis of petrobactin in 6 × 10^7^ spores of the wild type and *asb* mutant ± 25μM petrobactin (*n* = 1) harvested from ModG medium. (C and D) Oxidative stress survival by the wild type, the *asb* mutant strain ± 25μM petrobactin, and the *dhb* mutant strain grown in ModG medium for 8 h following incubation with either water (untreated) or 10 mM H_2_O_2_ for 10 min at 37°C. Samples were serially diluted and plated to calculate (C) the percentage of survival from (D) CFU/ml. Data were pooled from three independent experiments and analyzed using an unpaired *t* test. *, *P* < 0.05.

The petrobactin requirement for efficient sporulation and the upregulation of *asbA–F* during this period suggest that petrobactin is synthesized and may be present in the culture medium. However, the petrobactin-specific catechol moiety 3,4-dihydroxybenzoate was not detected in sporulation medium at 12 h postinoculation by the colorimetric catechol assay (data not shown). This could be due to either assay interference by the medium or petrobactin levels below the limit of detection or could suggest an intracellular role for petrobactin. To confirm petrobactin biosynthesis and address these possibilities, we used laser ablation electrospray ionization mass spectroscopy (LAESI-MS) to detect petrobactin in both the spent culture medium and the cell pellets of B. anthracis wild-type and *asb* mutant strains grown in sporulation medium for 12 h (previously validated in our lab [see reference 34]). Compared against our negative control, the petrobactin-null *asb* mutant strain, LAESI-MS confirmed the catechol assay results as it did not detect petrobactin in the spent culture medium from the wild-type strain (Fig. 4A), indicating no discernible export of this siderophore took place. However, petrobactin was detected in cells of the wild-type strain, thus confirming synthesis (Fig. 4A).

The use of petrobactin intracellularly might result in association of petrobactin with the B. anthracis Sterne spore, so we also subjected wild-type and *asb* mutant strain spores to LAESI-MS analysis (*n* = 3). This experiment detected petrobactin in wild-type but not *asb* mutant strain spores (Fig. 4B). This phenotype could be restored by supplementing growth and sporulation of the *asb* mutant strain with 25 µM purified petrobactin (*n* = 1). Complete ablation of the spores was confirmed by an abundance of the spore core component calcium dipicolinic acid in the chromatograph (data not shown). These data indicate that while petrobactin is not exported into the medium at detectable levels, it is biosynthesized but remains associated with the spore.

To this point in the studies, the mechanism of *asb* expression and role for petrobactin biosynthesis in sporulation remain unclear. Binding sites for *asbA–F* to regulators in the plasmid-based *asb* transcriptional reporter include PerR, an oxidative stress response regulator, and σ^B^, a general stress response regulator (Fig. 2A). In B. subtilis, σ^B^ is active during early sporulation, but is not required for either sporulation or an oxidative stress response, likely since most σ^B^-regulated genes can be activated by other transcription factors (40, 41). However, Lee et al. found that oxidative stress can induce petrobactin expression and synthesis, even under high-iron conditions (35). While sporulation is not known to be preceded by oxidative stress, B. subtilis cells become resistant to oxidative stress upon entry to the stationary phase (41–43). Additionally, oxidative stress protective enzymes are induced during late-stage growth of B. anthracis and maintained during sporulation, and two superoxide dismutases become incorporated in the exosporium (28, 29). Taken together with evidence of intracellular petrobactin, we predicted that petrobactin protects against oxidative stress.

To test this hypothesis, wild-type, asb mutant ± 25 µM petrobactin, and dhb mutant strains were tested for resistance to the oxidative stressor hydrogen peroxide (H_2_O_2_) at 8 h of growth (i.e., before sporulation) in sporulation medium. The percentage of survival was calculated by comparing the treated CFU/ml (those exposed to 10 mM H_2_O_2_) to untreated CFU/ml (water). While about 50% of the wild-type and the *dhb* mutant strain populations survived oxidative stress exposure, less than 1% of the *asb* mutant strain population survived (Fig. 4C). This was due to a 4-log decrease in the CFU/ml of the *asb* mutant strain following treatment with 10 mM H_2_O_2_ (Fig. 4D). The defect in survival was rescued by supplementation of the *asb* mutant strain with 25 µM purified petrobactin added to the medium at the time of inoculation (Fig. 4C and D). These data confirm the hypothesis that petrobactin is protective against oxidative stress during stationary phase—but prior to sporulation—in sporulation medium. This likely supports efficient sporulation and, in doing so, increases the number of viable spores and therefore transmission between mammalian hosts.

### Petrobactin is preferred for rapid growth and sporulation in bovine blood.

To determine potential roles for petrobactin influencing aspects of spore biology in a system nearer to what is seen in nature, bacterial growth and sporulation were followed in blood. The rationale for this is that following death of an infected mammal, blood laden with B. anthracis is exposed to the atmosphere by either hemorrhagic draining or the activity of scavengers on the carcass (11, 44, 45). Since vegetative bacilli are not easily infectious, B. anthracis transmission requires sporulation in aerated blood, a process triggered when the blood-borne CO_2_ reported to suppress sporulation decreases following death, thus triggering the sporulation cascade in a race against decomposition (44). Experiments to test *in vivo* sporulation are technically challenging, so to determine the relevance of each iron acquisition system—petrobactin, hemin, and bacillibactin—to disease, we measured sporulation in bovine blood. Cultures of wild-type B. anthracis Sterne, the *asb* mutant, the *dhb* mutant, and the *isd* mutant (with a mutation in hemin utilization) were grown in defibrinated bovine blood with shaking for 3 days. Every 24 h, the total and sporulated CFU/ml were enumerated.

**FIG 5:**
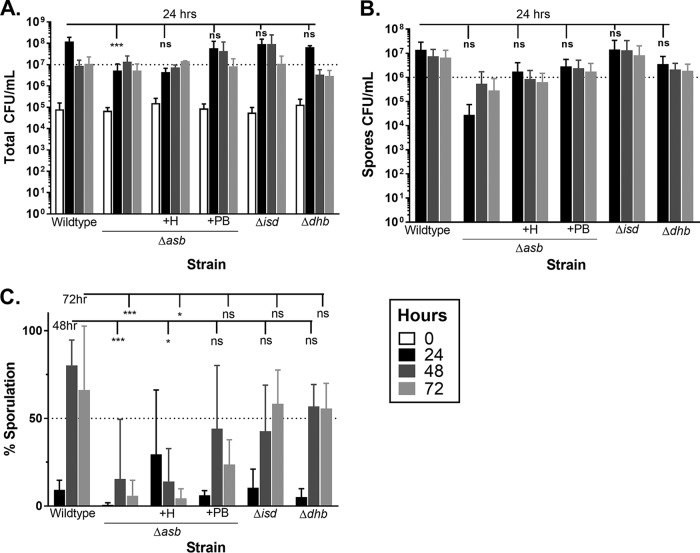
Petrobactin, but not hemin, is preferred for both growth and sporulation in bovine blood. The wild type, the *asb* mutant, the *isd* mutant, or the *dhb* mutant (supplemented with either 25 μM petrobactin [+PB] or 25 μM hemin [+H] as indicated) was grown in defibrinated bovine blood. At 24 (black), 48 (dark gray), and 72 (light gray) h postinoculation, CFU/ml were determined for both (A) total bacilli and (B) spores and used to calculate the (C) percentage of sporulation. Data were compiled from three independent experiments (except for the *asb* mutant plus 25 μM hemin [*n* = 2]) analyzed using a two-way analysis of variance (ANOVA) with a Tukey’s multiple-comparison test. ***, *P* ≤ 0.001; *, *P* < 0.05. ns, not significant. All other time point comparisons (e.g., 48-h wild-type versus 48-h *asb* mutant) were not significant. Dotted lines are placed to facilitate comparisons between strains and time points.

Compared to the wild type at 24 h, growth of the *asb* mutant strain was reduced by 1 log (Fig. 5A) with 2 log fewer spores (Fig. 5B), whereas all other strains—the *isd* and the *dhb* mutant strains—had equivalent CFU/ml. While the percentage of sporulation at 24 h is low, generally <25%, most sporulation in the wild-type strain appears to occur during the first 24 h of incubation, after which nonsporulated cells begin to die, thus reducing the total cells and increasing the percentage of spores. Conversely, the *asb* mutant strain demonstrated delayed sporulation, gaining an additional log of spores between the 24- and 48-h time points, but the percentages of sporulation at 48 and 72 h were <25% compared to the wild-type strain at 80% (Fig. 5C). The partial rescue of *asb* sporulation by petrobactin supplementation (increasing CFU and spore counts, but not percentage of sporulation) could be the result of external petrobactin supplementation: perhaps intracellular petrobactin is required to fully protect against host oxidative stress. The percentages of sporulation for both the *isd* mutant strain and the *dhb* mutant strain were about 50%, although these were not statistically significantly different from the wild-type strain (Fig. 5C). Additionally, both total and spore CFU/ml for both the *isd* and *dhb* mutant strains were like those of the wild type, suggesting that petrobactin is a preferred iron-gathering system during growth in bovine blood (Fig. 5A and B).

The growth defect and delayed sporulation of the *asb* mutant strain could be due to oxidative stress, a lack of available iron, or a combination of the two stresses. To separate the effects of petrobactin supplementation on iron acquisition and protection from oxidative stress, the *asb* mutant strain was supplemented with 25 µM of either petrobactin or hemin (*n* = 2). Hemin is the oxidized form of heme, which is released into blood by the lysis of red blood cells and can be bound by B. anthracis hemophores, making it a biologically relevant iron source (46, 47). However, hemin is not known to protect against intracellular oxidative stress, so we predicted that if petrobactin were only required for iron acquisition, then hemin supplementation should complement the *asb* mutant strain sporulation phenotype.

Supplementation of the *asb* mutant with hemin did not affect overall growth but appeared to enhance early sporulation, whereas supplementation with petrobactin rescued both growth and sporulation (Fig. 5A to C). These data suggest that the iron provided via hemin may allow for efficient sporulation, while the dual benefits of petrobactin iron acquisition plus protection from oxidative stress enable continued growth prior to the onset of sporulation.

## DISCUSSION

In this work, we show that petrobactin is not required for B. anthracis Sterne germination (Fig. 1A) but is required for efficient sporulation in sporulation medium (Fig. 1B). Using fluorescent *asbA*::*gfpmut3*α reporter fusions, we also show that *asb* is both transcribed and translated during late-stage growth of B. anthracis Sterne prior to sporulation in a sporulation sigma factor-independent manner (Fig. 2B and C and Fig. 3). Unlike during vegetative growth, petrobactin is not exported during sporulation but remains intracellular (Fig. 4A), where it has a statistically significant role in protecting against oxidative stress (Fig. 4C and D) and eventually associates with the spore (Fig. 4B). These findings may have relevance to transmission since petrobactin is also required for efficient sporulation in bovine blood (Fig. 5), a prerequisite for survival and transmission of the pathogen (11, 44). We believe this to be the first demonstration that a siderophore is induced in preparation for sporulation and present in the mature spore.

The iron-gathering capacity of siderophores has long been appreciated for their role in pathogenicity, and since their discovery, evidence for alternate functions has accumulated. Multiple reports have demonstrated roles for siderophores in cell signaling, sporulation initiation, protection from copper and oxidative stresses, and the generation of oxidative stress against competitors, as well as most recently, in survival via spores (48, 49).

In early 2017, Grandchamp et al. showed with B. subtilis that siderophore supplementation (including with the native bacillibactin) caused the onset of sporulation to occur earlier (49). Since this enhancement required import of the siderophore into the bacterial cell and iron removal by corresponding hydrolases, these authors hypothesized that the extra intracellular iron acted as a signal for the onset of sporulation (49). However, their study did not address bacillibactin regulation, export during sporulation, or the cell stresses associated with sporulation. So, to our knowledge, this article is the first demonstration that a siderophore is induced to protect against oxidative stress prior to sporulation under high-iron conditions.

As noted in the introduction, siderophores are primarily regulated by the iron-dependent repressor Fur. However, some siderophores, such as petrobactin, are biosynthesized in response to oxidative stress conditions and other catecholate-containing siderophores (e.g., enterobactin and salmochelin) are protective against reactive oxygen species (35, 50–55). This protection is not due to iron sequestration that prevents additional Fenton reactions but is a function of the antioxidant properties of catechols (50, 53). Supplementation with free catechols does not rescue the protective function of enterobactin, which requires import and hydrolysis for effective oxidative stress protection (53, 54). It is unclear whether petrobactin requires additional processing to become active against oxidative stress, though the detection of petrobactin associated with spores by mass spectrometry suggests it does not.

There are at least two nonexclusive hypotheses for siderophore upregulation during oxidative stress: (i) that superoxide radicals oxidize iron cofactors, thus inactivating key enzymes, and (ii) that upregulation of the enzymes to mitigate oxidative stress requires metallic (e.g., iron and manganese) cofactors. Both of these would reduce the intracellular iron pool and thereby relieve iron from Fur to enable iron acquisition system expression (35, 51). In the case of Bacillus spp., the intracellular iron pool is further depleted during the onset of sporulation due to upregulation of aconitase, an iron-rich citrate isomerase and stabilizer of σ^K^-dependent gene transcripts (56, 57).

While the demand for iron during oxidative stress and/or sporulation may relieve negative regulation by Fur, it is likely that *asbA*–*F* expression is induced by an oxidative stress regulator such as PerR. Enterobactin is positively regulated by the oxidative stress response, and there is compelling evidence linking Azotobacter vinelandii catecholate siderophores to similar regulation (53–55). The observed phenotype for those siderophores is similar to that observed by Lee et al. for petrobactin: high-iron repression of the siderophore can be overcome by oxidative stress (35, 53, 55). While oxidative stress has not been directly linked to sporulation, many stressors (heat, pH, and antibiotics) induce secondary oxidative stress in Bacillus spp. by perturbing the electron transport chain (42). Additionally, several oxidative stress protective enzymes are already upregulated during sporulation, suggesting concomitant activity by PerR, which is upregulated prior to sporulation (28). Petrobactin biosynthesized within the cell to protect against such oxidative stress may then become randomly associated with the prespore. More work is needed to better characterize regulation of both the *asb* operon, particularly by PerR, and petrobactin biosynthesis.

The early experiments establishing that petrobactin is required for B. anthracis pathogenesis involved germination and growth in macrophages, which may be another source of oxidative stress within the mammalian host. B. anthracis spores both germinate and outgrow within the phagolysosomes of APCs, in which they are bombarded with host-derived stressors, such as antimicrobial peptides, low pH, and oxidative species (3, 58). The oxidative stress protective enzymes and petrobactin, incorporated into the spore during sporulation, may also protect under these conditions (29).

As growth in blood marks an endpoint for an anthrax infection, the bacilli must not only grow well but also prepare for survival and transmission between hosts. Evidence in the literature suggests that exposure of blood-borne bacilli to oxygen as a dying host bleeds out begins the signaling cascade for sporulation, creating a direct link between growth and sporulation in blood and transmission (11, 44). It is known that petrobactin is required for growth in macrophages and iron-depleted medium, but that requirement had not been demonstrated for growth or sporulation in blood prior to these experiments. Our data suggest that petrobactin is the preferred iron acquisition system for growth and sporulation in bovine blood, despite multiple potential iron sources. While, petrobactin was required to achieve wild-type growth of 10^8^ CFU/ml in blood, the *asb* mutant was still able to grow to 10^7^ CFU/ml, suggesting that another iron acquisition source was functioning—likely either the *isd* system or bacillibactin. More work is needed to fully understand the contributions of each iron system to growth and sporulation and to verify these findings in other B. anthracis strains.

**FIG 6:**
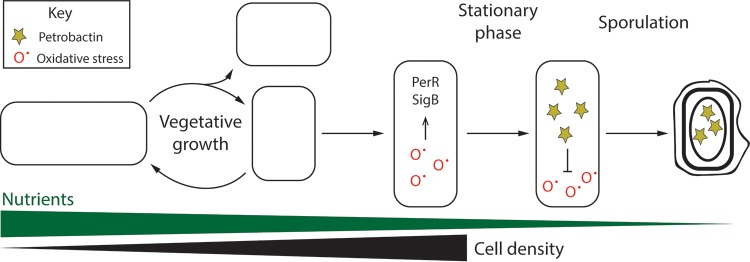
Proposed model of petrobactin use by B. anthracis during late-stage growth and early sporulation.

These data update the model of B. anthracis Sterne iron acquisition and sporulation (Fig. 6). In this model, upon entry of the bacterial population into late-stage growth, environmental stressors both deplete the intracellular iron pool and induce oxidative stress, which act to upregulate the *asb* operon, presumably through PerR regulation. Petrobactin is biosynthesized for iron acquisition and/or protection against oxidative stress, which support the bacillus as it transitions into sporulation. Either direct import of petrobactin into the prespore or random association results in packaging of petrobactin into the spore. These findings underscore the vital role of petrobactin in the many stages of B. anthracis infection, from survival in the macrophage to growth in the bloodstream and, now, sporulation, which facilitates transmission to a new host.

## MATERIALS AND METHODS

### Bacterial growth conditions and sporulation.

The strains used in this study are described in Table S1 in the supplemental material. Genomic-based fluorescent reporters were generated by PCR amplification and Gibson cloning (New England Biolabs) of the genetic construct into pBKJ258, which was then inserted onto the Bacillus anthracis Sterne 34F2(pXO1^+^, pXO2^−^) genome by allelic exchange, as described by Janes and Stibitz (59). The plasmid-based transcriptional reporter was directionally cloned with EcoRI and BamHI into the pAD123 multiple cloning site upstream of the promoterless *gfpmut3*α. All necessary primers are listed in Table S2 in the supplemental material. Modified G medium (ModG) was used for the generation of B. anthracis spores at 37°C for 72 h (60). Spores were collected at 2,800 rpm and then washed and stored in sterile water at room temperature following heat activation at 65°C. Strains containing plasmid-based reporters (61) were grown in the presence of 10 μg/ml chloramphenicol. Media and chemicals were purchased from Fisher Scientific or Sigma-Aldrich.

10.1128/mBio.02079-18.2 TABLE S1 Strains of B. anthracis Sterne 34F2 used in this work. Download Table S1, PDF file, 0.1 MB. Copyright © 2018 Hagan et al. 2018 Hagan et al. This content is distributed under the terms of the Creative Commons Attribution 4.0 International license.

10.1128/mBio.02079-18.3 TABLE S2 Primers used to generate mutant strains used in this work. Download Table S2, PDF file, 0.1 MB. Copyright © 2018 Hagan et al. 2018 Hagan et al. This content is distributed under the terms of the Creative Commons Attribution 4.0 International license.

### Spore germination.

Spore germination was measured in iron-depleted medium (IDM) supplemented with 1 mM inosine, following a 20-min heat activation at 65°C (30). To measure germination and subsequent outgrowth, spores were inoculated at a starting OD_600_ of between 0.25 and 0.5 for a final volume of 200 µl (*n* = 3). The spores were incubated at 37°C in a SpectraMAX M2 spectrophotometer, and the OD_600_ was measured every 5 min for 1 h. Data are representative of three independent experiments and are presented as a a percentage of the initial OD_600_.

### Supplementation of *asb* mutant sporulation with petrobactin.

To supplement *asb* mutant spores with petrobactin, bacilli were grown overnight at 30°C in brain heart infusion (BHI [Difco]) inoculated 1:1,000 in 25 ml of ModG medium supplemented with 25 µM purified petrobactin. After a 72-h incubation, spores were collected by centrifugation at 2,800 rpm and washed three times with 20 ml of sterile, deionized water. The spores were resuspended in 1 ml of water following heat activation for 20 min at 65°C.

### Reporter growth, measurement, and analysis.

Bacterial strains were plated on BHI and grown in BHI at 30°C overnight. Overnight cultures were back-diluted 1:50 into fresh BHI and incubated at 37°C for 1 h. The cells were pelleted at 2,800 rpm for 10 min (Eppendorf 5810 R centrifuge), washed once with BHI, and then used to inoculate 200 µl of ModG medium. Each strain was inoculated into triplicate wells of a 96-well plate to a starting OD_600_ of 0.05, covered with a gas-permeable sealing membrane (Breathe-Easy; Diversified Biotech), and then grown in a Synergy HTX plate reader at 37°C with continuous shaking at 237 cpm for 12 h. The OD_600_ and fluorescence (excitation, 485/20; emission, 528/20) were bottom read every 5 min using a tungsten light source. Data were analyzed in R software by first subtracting a medium blank from both fluorescence and OD_600_ and then normalizing fluorescence by the OD_600_ (62). Background fluorescence was approximated by wild-type cells and subtracted from the reporters at corresponding time points.

### Microscopy.

Cells of the wild-type strain and the strain containing the *asb* translational reporter expressing Gfpmut3α were grown in ModG medium at 37°C, and every 2 h from 6 to 12 h postinoculation, 5 µl was spotted onto a microscope slide. At least 100 bacteria were imaged at each time point and scored for fluorescence and/or sporulation. Bacteria were counted by determining the size of a bacterium and calibrating all images to this length. A bacterium was scored as positive for fluorescence if the intensity was at least 1.4× above the background fluorescence of wild-type, non-Gfpmut3α-expressing bacilli. A bacterium was positive for sporulation upon observation of a phase-bright spore. Phase-contrast and fluorescence microscopic images were taken using a Nikon TE300 inverted microscope equipped with a mercury arc lamp, 60× Plan-Apochromat 1.4-numerical aperture objective, cooled digital charge-coupled device (CCD) camera (Quantix Photometrics). Excitation and emission wavelengths were selected using a 69,002 set (Chroma Technology) and a Lambda 10-2 filter wheel controller. Fluorescence images of Gfpmut3α were captured with excitation and emission filters centered at 490 nm and 535 nm, respectively. Exposures were set at 300 ms.

### Oxidative stress survival.

Wild-type, *asb* mutant, and *dhb* mutant strains were grown in ModG medium. At 8 h postinoculation, 500 µl of each culture was added to 100 µl of either sterile water or 60 mM hydrogen peroxide (final concentration, 10 mM). Treated (10 mM H_2_O_2_) and control (H_2_O) cultures were incubated for 10 min at 37°C and then serially diluted in phosphate-buffered saline (PBS) and plated on BHI to stop the reaction and count CFU/ml. Any culture below the limit of detection (about 667 CFU/ml) was assigned a conservative value of 600 for data analysis. Data are pooled from three independent experiments and presented as % survival = (treated/untreated) × 100.

### LAESI-MS.

Samples from ModG medium for LAESI-MS were collected at 12 h postinoculation and separated by centrifugation to obtain the culture medium and cell pellets. Cells were washed once in an equal volume of PBS. All samples were stored at −80°C until analysis. Spores for LAESI-MS analysis were prepared as described above. Unless indicated otherwise, 6 × 10^7^ spores from three independent spore preparations in 50% dimethyl sulfoxide (DMSO) (or 15 µl of cell pellets) were plated in triplicate wells of shallow 96-well plates and subjected to laser-based ablation. Data were collected and analyzed as previously described (34). The average intensity of petrobactin was normalized as necessary (e.g., OD_600_ or 10^6^ spores).

### Sporulation efficiency.

The wild-type and *asb*, *dhb*, and *isd* mutant strains were grown in BHI overnight at 30°C and then inoculated at 1:1,000 into 3 ml of either ModG medium or defibrinated bovine blood (Hemostat Laboratories). Cultures were grown at 37°C. Total and sporulated CFUs were enumerated at regular intervals by serial dilution in PBS prior to plating on BHI for growth at 37°C overnight. Cultures were plated both before and after a heat treatment step (30 min at 65°C) to obtain total cells and spores, respectively. CFU/ml below the limit of detection (∼667) were assigned a conservative value of 600 for data analysis. % sporulation = (post-heat treatment/total) × 100. Hemin for supplementation was first suspended at 3.83 mM in 1.4 M NaOH and then diluted to 150 µM in PBS (63). Data were pooled from three independent experiments unless otherwise noted.

10.1128/mBio.02079-18.1 FIG S1 Phase-contrast and fluorescent imaging of wild-type B. anthracis Sterne bacilli during growth in sporulation medium. The strain was grown in ModG sporulation medium with both phase-contrast and fluorescent (excitation, 490 nm; emission, 535 nm) micrographs taken at 6, 8, 10, and 12 h of growth. Representative images from each time point are shown. (A) Six hours. (B) Eight hours. (C) Ten hours. (D) Twelve hours. Download FIG S1, PDF file, 1.6 MB. Copyright © 2018 Hagan et al. 2018 Hagan et al. This content is distributed under the terms of the Creative Commons Attribution 4.0 International license.
