# Supplementary material for: Petrobactin Protects against Oxidative Stress and Enhances Sporulation Efficiency in Bacillus anthracis Sterne
Source: mBio. 2018 Nov 6;9(6):e02079-18. doi: 10.1128/mBio.02079-18 (PMC6222121; doi:10.1128/mBio.02079-18)
Supplement: TABLE S1 [file mbo005184145st1.pdf]

Supplementary Table 1. Strains of *B. anthracis* Sterne 34F2 used in this work.

Strain	Relevant characteristics	Reference
Bacillus anthracis Sterne 34F2	Wild-type ($pXO1^+$, $pXO2^-$)	Sterne, 1939
34F2, ΔasbABCDEF	Petrobactin biosynthesis mutant	Lee et al., 2007
34F2, Δdhb	Bacillibactin biosynthesis mutant	Cendrowski et al., 2004
34F2, Δisd	Heme import system mutant	This work
34F2, BA140	*asbA:gfpmut3α* transcriptional fusion	This work
34F2, BA141	*asbA:gfpmut3α* translational fusion	This work
34F2, SC140	promoterless *gfpmut3α* (pAD123)	This work
34F2, SC136	asb^P*:gfpmut3α* transcriptional fusion (pSC118)	This work
