# Supplementary material for: Petrobactin Protects against Oxidative Stress and Enhances Sporulation Efficiency in Bacillus anthracis Sterne
Source: mBio. 2018 Nov 6;9(6):e02079-18. doi: 10.1128/mBio.02079-18 (PMC6222121; doi:10.1128/mBio.02079-18)
Supplement: TABLE S2 [file mbo005184145st2.pdf]

Supplementary Table 1. Primers used to generate mutant strains used in this work.

Primer	Sequence (5'→3')
Isd P1	GCTAAAATTACAAAGCATACATACGAACGTTATAATAAAAGCGG
Isd P2	CGTTCGTATGTATGCTTTGTAATTTTAGCTGTATTTGTAACAATAATC
Isd P3	GGAACAAAAGCTGGAGCTCCACCGCGGTGGCCTCCAAGCACCTCTTTTGGATTAAT
Isd P4	GGATATCAGATCTGACGTCTCTAGAGCGGCCGCTTTCTATAATAGAAAGTCTCTTTTTTTGTAAAA
Isd P5	GTGATACGAGTGGACTAAAAAC
Isd P6	CTGTAGAAAAATAGAGAAAAGAG
asb^{P}:gfpmut3α + RBS R	ACCTCCTTTAACAGGTTATGTAACGTAATCTGCTTTCATAACAGAATTAC
asb^{P}:gfpmut3α R	TTTACTAGATCTCATTGTAACGTAATCTGCTTTCATAACAGAATTA
gfpmut3α + RBS:asb^{P} F	AGATTACGTTACATAACCTGTTAAAGGAGGTGTTTTCTAGAATG
gfpmut3α F	GATTACGTTACATAAATGAGATCTAGTAAAGGAGAAGAACTTTTCAC
gfpmut3α R	CGATTTCTAGCCATTTTATTTGTATAGTTCATCCATGCCATGT
asb^{P}: gfpmut3α diagnostic F	GCATCACCTTCACCCT
asb^{P}_P1	GAACTATACAAATAAAATGGCTAGAAATCGTAATTCTAATCAATTAG
asb^{P}_P2	ACAATTGTATGTACCTTAAGAAGTTACTACTTCTAAATTTCTATTTGTTAGTACTTT
asb^{P}_P3	GGAACAAAAGCTGGAGCTCCACCGCGGTGGCCAACAAGAGAGACAAGAGTATGTGAATC
asb^{P}_P4	GATATCAGATCTGACGTCTCTAGAGCGGCCTCATAACAAGTGGAACTGATGCAAGC
asb^{P} F	GTAGTAACTTCTTAAGGTACATACAATTGTGAGGGAGAATTATATG
asb^{P} + RBS:gfpmut3α R	TAGTGTATCAATTCATTATGTAACGTAATCTGCTTTCATAACAGAATTAC
asb^{P} R	GTCAGTAACTTCCACTGTAACGTAATCTGCTTTCATAACAGAATT
asb^{P}:gfpmut3α diagnostic R	GGAACCATTACCATATTTCTC
SC140 F	GAGGTATACCTCTTTTGTTTAACTATTTTGG
SC140 R	CCGATAGTGTTTATAGAAAGAGGAGGGCAAGTA
