# Supplementary figures and images for: Petrobactin Protects against Oxidative Stress and Enhances Sporulation Efficiency in Bacillus anthracis Sterne
Source: mBio. 2018 Nov 6;9(6):e02079-18. doi: 10.1128/mBio.02079-18 (PMC6222121; doi:10.1128/mBio.02079-18)

**Phase Contrast**

**Fluorescent**

**A.**

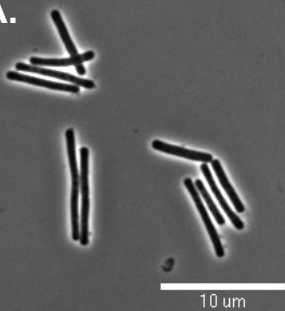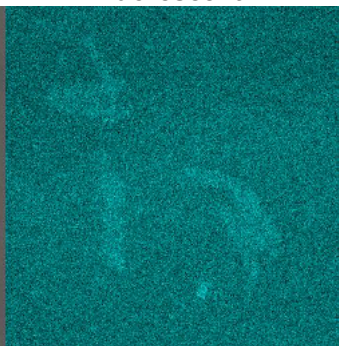

**B.**

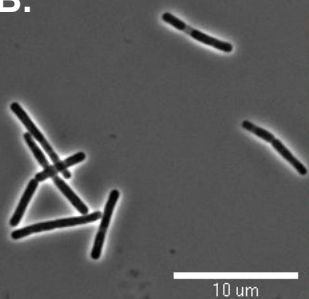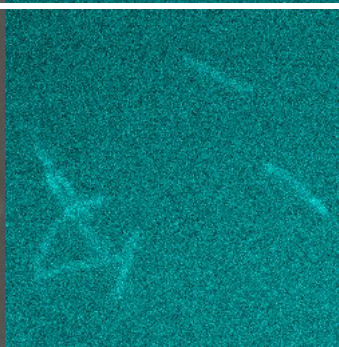

**C.**

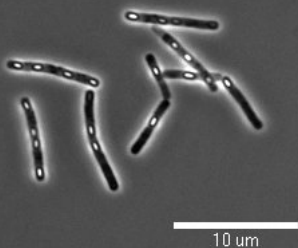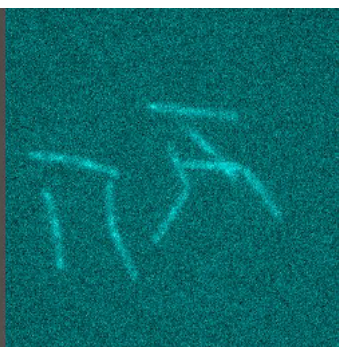

**D.**

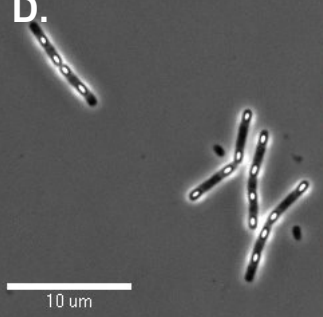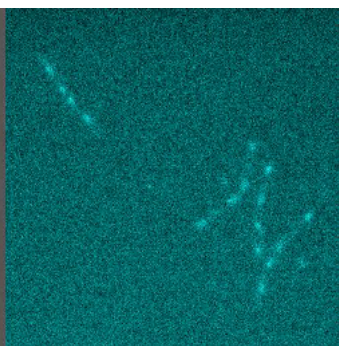

Supplement: FIG S1 [file mbo005184145sf1.pdf]
